# The valance state of vanadium-key factor in the flexibility of potassium vanadates structure as cathode materials in Li-ion batteries

**DOI:** 10.1038/s41598-022-23509-x

**Published:** 2022-11-05

**Authors:** M. Prześniak-Welenc, M. Nadolska, K. Jurak, J. Li, K. Górnicka, A. Mielewczyk-Gryń, M. Rutkowska, A. P. Nowak

**Affiliations:** 1 Institute of Nanotechnology and Materials Engineering, Gdansk University of Technology, Narutowicza 11/12, 80-233 Gdansk, Poland; 2 Faculty of Chemistry, Gdansk University of Technology, Narutowicza 11/12, 80-233 Gdansk, Poland; 3 Key Laboratory for Thin Film and Microfabrication of Ministry of Education, Department of Micro/Nano-Electronics, Shanghai Jiao Tong University, Shanghai, 200240 China

**Keywords:** Batteries, Nanoparticle synthesis

## Abstract

Potassium hexavanadate (K_2_V_6_O_16_·nH_2_O) nanobelts have been synthesized by the LPE-IonEx method, which is dedicated to synthesis of transition metal oxide bronzes with controlled morphology and structure. The electrochemical performance of K_2_V_6_O_16_·nH_2_O as a cathode material for lithium-ion batteries has been evaluated. The KVO nanobelts demonstrated a high discharge capacity of 260 mAh g^−1^, and long-term cyclic stability up to 100 cycles at 1 A g^−1^. The effect of the vanadium valence state and unusual construction of the nanobelts, composed of crystalline and amorphous domains arranged alternately were also discussed in this work. The ex-situ measurements of discharged electrode materials by XRD, MP-AES, XAS and XPS show that during the subsequent charge/discharge cycle the potassium in the K_2_V_6_O_16_·nH_2_O structure are replacing by lithium. The structural stability of the potassium hexavandate during cycling depends on the initial vanadium valence state on the sample surface and the presence of the “fringe free” domains in the K_2_V_6_O_16_·nH_2_O nanobelts.

## Introduction

Over the past decades, vanadium-based oxides^1–8^, and vanadium bronzes, in particular vanadium oxide bronzes^9–12^ have been extensively studied, due to their high specific capacity, low cost, and better safety characteristics, as potential cathode materials for rechargeable lithium-ion batteries. Most vanadium bronzes with the general formula A_x_V_y_O_z_, where A is virtually any cation of an alkali or earth alkali metal, have a stable structure for intercalation of Li^+^ and Na^+^. Therefore, they have been applied to non-aqueous and aqueous LIBs or Sodium-ion batteries^13,14^, especially nanostructured form. The nanostructural morphology can shorten the diffusion path of ions and increase the contact area between the electrolyte and active materials^15^. The potassium vanadates are considered as positive electrode materials for LIBs since the 1980s when Raistrick and Huggins for the first time examined their electrochemical performance^16,17^. Since then, many potassium vanadates compounds have been investigated as cathode materials. Manev et al. have reported the electrochemical performance of series of potassium vanadates such as KV_3_O_8_, K_2_V_8_O_21_, K_3_V_5_O_14_, and KV_5_O_13_, and among them, the last one exhibits the best specific capacity of about 210 mAh g^−1^, and good cyclability^18^. Baddour-Hadjean et al.^19^, investigated the influence of local structure on electrochemical performance of KV_3_O_8_ and K_0.5_V_2_O_5_ with layered structures, and K_0.25_V_2_O_5_ with a tunnel framework isomorphic structure. They demonstrated, that the potassium-richer compound KV_3_O_8_ has good rechargeability despite a low discharge capacity (70 mAh g^−1^), while the potassium-poorer bronze K_0.25_V_2_O_5_ exhibits the high specific capacity (230 mAh g^−1^) but slow and continuous capacity fade with cycling, meanwhile the K_0.5_V_2_O_5_ combined the remarkable specific capacity 210 mAh g^−1^ with excellent capacity retention. It should be underlined, that the present studies of potassium vanadates electrochemical performance are mostly focus on K_0.25_V_2_O_5_^20–23^ and K_0.5_V_2_O_5_^24–26^ in hydrated or non-hydrated form_._ Recently, the K_2_V_6_O_16_·nH_2_O has attracted much attention as positive electrode material in metal-ion batteries. The crystal structure of potassium hexavanadates can be described as a hewettite type structure where the layered V_3_O_8_ backbone is formed by two basic structural units of VO_6_ and V_2_O_8_ polyhedrons. In addition, the hydrated K ions are located between the layers, which stabilize the structure and facilitate intercalation/deintercalation of guest ions and act as “pillars”^27–30^. Furthermore, water molecules inside the potassium hexavanadate expand the interlayer distance, and promote ion diffusion^31^. Above mentioned features led to studies of K_2_V_6_O_16_·nH_2_O as a cathode material for calcium-ion^27^ or zinc-ion^28,32^ batteries. However, to the best of our knowledge, it has never been considered for lithium-ion batteries.

In this work, a comprehensive study of K_2_V_6_O_16_·nH_2_O as a cathode material for LIBs is reported for the first time. We investigated the influence of the vanadium valence state on the electrochemical performance in two potential ranges (2.0–4.0 V and 1.5–4.0 V). The hydrated potassium vanadate was obtained by an innovative method, based on the Liquide-Phase Exfoliation with Ion Exchange (LPE-IonEx)^33^. This method is dedicated to the synthesis of transition metal oxide bronzes with controlled morphology and structure, moreover opposite to the other synthesis method, allows obtain the single-phase compounds. The stability of potassium hexavanadate was explored based on ex-situ characterization involving the phase as well as the change in vanadium valence after cycling performance. We have demonstrated that the valence state of vanadium on the sample surface plays a crucial role in the structural flexibility and electrochemical performance of K_2_V_6_O_16_·nH_2_O. Moreover, this compound is a promising candidate for a positive electrode for rechargeable lithium batteries and exhibits a remarkable specific capacity of 260 mAh g^−1^ combined with excellent capacity retention (> 99% after 100 cycles) among the potassium–vanadium bronzes.

## Experimental

### Synthesis

Potassium formate (99%, Sigma Aldrich), V_2_O_5_ (99.2%, Alfa Aesar) were used as reagents without further purification. Samples were prepared via the LPE-IonEx method. To 50 mL of 1 M solution of potassium formate in deionized water (DIW), 500 mg of V_2_O_5_ was added. The mixture was vigorously stirred for 48 h at two temperatures: RT (sample KVO-20) and 40 °C (sample KVO-40). From the initial solution, rusty red solid residues were collected by centrifugation. After washing several times with DIW, the product was dried overnight at 40 °C under reduced pressure (0.01 bar).

### Characterization

X-ray diffraction patterns (XRD) were collected in the range 2θ of 5°–70° by a D2 Phaser diffractometer (Bruker) with CuK_α_ radiation (λ = 1.5404 Å). The FullProf Suite program was used to perform Le Bail refinements using Thompson–Cox–Hastings pseudo-Voigt peak shapes. The Fourier transform infrared (FTIR) spectra were recorded in the range of 400–4000 cm^−1^ on Perkin Elmer Frontier spectrophotometer. The surface morphology and fine grain structure were studied by a FEI Company Quanta FEG 250 scanning electron microscope (SEM) and an FEI TECNAI G2 F20 transition electron microscopy (TEM). Nitrogen adsorption–desorption isotherms were measured on a surface area analyser NOVAtouch™ 2 (Quantachrome Instruments) at 77 K. Prior to the measurements, samples were degassed at 40 °C for 12 h under dynamic vacuum. The specific surface area was calculated using the Brunauer–Emmett–Teller (BET) linear equation in the relative pressure range (p/p_0_) from 0.1 to 0.3 from six points. The correlation coefficient of the linear regression was not less than 0.99. Thermogravimetric analysis (TG) was carried out under argon atmosphere with a flow rate of 60 ml/min in the temperature range 40–350 °C (heating rate 5 °C/min) using STA 449 F1 (Netzsch). The high-resolution X-ray photoelectron spectroscopy (XPS) analysis was performed using an Escalab 250Xi device (ThermoFisher Scientific), equipped with a mono-chromatic AlKα source. Measurements were carried out at 25 eV pass energy with 0.05 eV energy step size. The X-ray spot size was 250 µm. The calibration of the XPS spectrum was done using the characteristics peak of adventitious carbon C1s at 284.6 eV^34^. The X-ray absorption (XAS) measurements were performed at the ASTRA beamline at SOLARIS National Synchrotron Radiation Centre, Cracow, Poland. The V K-edge XANES of powder samples and after-cycled electrodes were obtained in transmission mode in the range 5265 to 5550 eV. Concentration of the elements in the samples determined using atomic emission spectrometry with microwave plasma atomisation (MP-AES) supplied by Agilent. K, Li, V standard solution were obtained from Sigma Aldrich, Ms Spectrum and J.T. Baker, respectively. The determination of the elements in the samples was carried out at specific wavelengths (Li—670.784 nm, K—766.491 nm, and V—437.923 nm).

### Electrochemical measurements

The electrodes were prepared from a slurry containing a 7:2:1 weight ratio of KVO-20 or KVO-40, carbon black (Super P^®^, Timcal) as conducting agent, and 10 wt% polyvinylidene fluoride (Solef^®^ 6020) as a binder in N-methylpyrrolidone (AlfaAesar). The slurry was spread on the aluminum foil. The foil was dried under a dynamic vacuum in an oven (Glass Oven B-595, Büchi) for 24 h at 90 °C. Next, the discs of 10 mm diameter were cut from the film. The average loading mass of electrode material was ~ 0.5 mg. Dried disc electrodes were used in two-electrode pouch cells with lithium foil as a counter and reference electrodes (99.9% purity, 0.75 mm thickness, Alfa Aesar) in 1 M LiPF_6_ in EC:DMC ratio 1:1 (LP30 Merck) as the electrolyte, glass fiber (Schleicher & Schüll) as the separator, and polyester film as an adhesive tape (RD-697, PPI, Ireland). The battery tests, including galvanostatic polarization as well as cyclic voltammetry were performed using the ATLAS 0961 MBI (Poland) multichannel battery testing system within the potential range from 1.5 to 4.0 V, and from 2.0 to 4.0 V vs. Li/Li^+^ with a scanning rate of 0.01, 0.02, 0.05 and 0.1 mV s^−1^. Galvanostatic intermittent titration technique (GITT) was applied after for the first charge/discharge cycle with a current density of 100 mA g^−1^, pulse duration of 1800s and 2 h relaxation time. The cut-off potential was 1.5 V for charging and 4.0 V for discharging.

## Results and discussion

### Structural analysis

**Figure 1:**
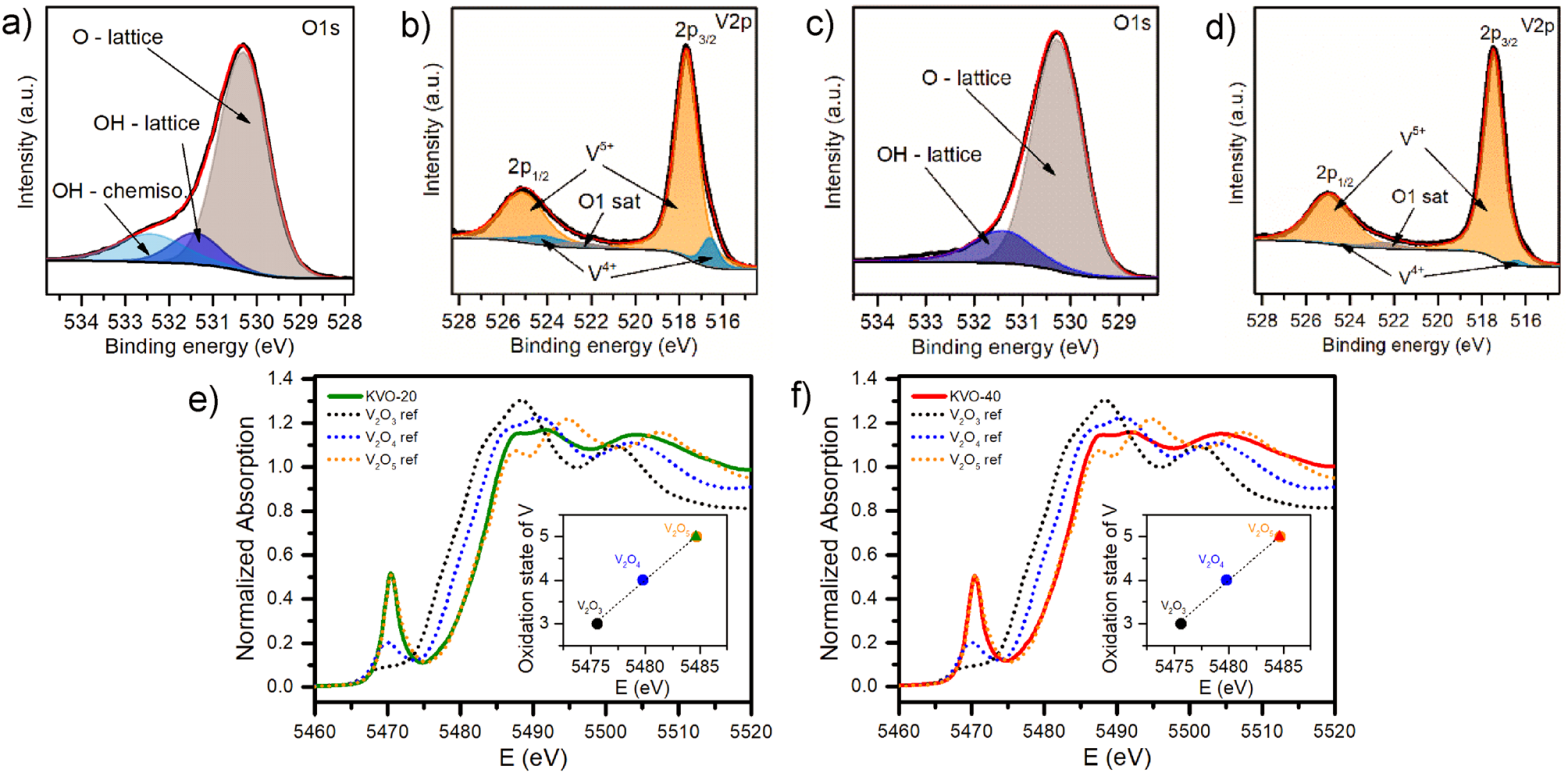
(**a–d**) High resolution XPS spectra of the O 1s and V 2p regions for KVO-20 (**a,b**) and KVO-40 (**c,d**), (**e,f**) V K-edge the XANES for KVO-20 (**e**) and KVO-40 (**f**), respectively (the insets show the relation between vanadium oxidation state and the edge position).

The XRD patterns of sample KVO-20 and KVO-40 (Fig. S1, ESM) can be indexed within the monoclinic K_2_V_6_O_16_·1.5H_2_O phase (JCPDS no. 00-051-0379). The lattice parameters, determined from Le Bail refinement, are given in Supporting Information (Fig. S2, Table S1, ESM).To confirm the lattice water presence in the samples, TG analysis was performed (Fig. S3). The weight loss between 40 and 100 °C is attributed to the evaporation of physisorbed water and between 100 and 350 °C to chemisorbed structural water^14^. The lattice water attributed loss for KVO-20 is 1.75 wt% and 2.05 wt% for KVO-40, which corresponds to 0.65 and 0.76 molecules of water per K_2_V_6_O_16_ formula unit, respectively. The FTIR spectra of the samples are shown in Fig. S4 in the ESM. The bands at ~ 1005 and ~ 970 cm^−1^ can be assigned to V=O vibrations, while those at 525 and 590 cm^−1^ to the symmetric and asymmetric stretching of V–O–V^30^. The band at 730 cm^−1^ corresponds to bridging V–O···K stretching^35^. The band at 950 cm^−1^ visible for both samples, suggests the existence of the OH bridge between two metals, likely vanadium^36^. In addition, bands related to the water molecules (stretching and bending vibrations at ∼ 3450 cm^–1^ and ∼ 1620 cm^–1^, respectively) can be observed for both samples^30^. Next, the XPS technique was used to investigate the chemical composition and the valence state of vanadium ions in the samples. The O 1s core-level spectra show deconvoluted peaks (530–532 eV), three for sample KVO-20 (Fig. 1a), and two for KVO-40 (Fig. 1c). The lower binding energy at 530.3 eV corresponds to O–lattice^27^. The second peak centered at 531.4 eV is attributed to the loosely bounded oxygen ions on the surface, namely, OH^−^ groups^37^. The third peak centered at 532.5 eV, observable only for sample KVO-20, is ascribed to chemisorbed OH^−^ ions on the surface^38^. The XPS for V 2p (Fig. 1b,d) shows two peaks located around 525 and 517.7 eV, which are attributed to V 2p_1/2_ and V2p_3/2,_ respectively. The V2p_3/2_ band for both samples shows asymmetry shaped with a very weak shoulder line on the lower binding energy side, two different components of V2p_3/2_ are apparent. The strong peak around 517.5 eV and the weak peak around 516.5 eV were corresponding to the V^5+^ and V^4+^ respectively^39^. These components are also present for V 2p_1/2_ peak, the two peaks centered at 525 eV and 524 eV were assigned to the V^5+^ and V^4+^,respectively (the Fig. S5a,b, ESM). The peak located around 522 eV was attributed to the O1s satellite^40^. Based on the area of the fitted curves, the relative atomic ratio of V^4+^/V^5+^ present on the sample surface were estimated to be 0.10 for sample KVO-20 and 0.02 for sample KVO-40. The surface vacancies interact with the H_2_O molecules, which leads to water chemisorption^41^. Therefore, due to the 5-times smaller concertation of V-vacancy on the KVO-40 sample surface, the peak centered at 532.5 eV (chemisorbed OH^−^ ions) is very likely to be overlapped with the peak located at 531.4 eV (OH–lattice).

The XPS spectrum in K 2p core level binding energy (Fig. S5c,d, ESM) show two characteristic peaks of the 2p_3/2_ and 2p_1/2_ spin–orbit of K^+^ coupled energy states, centered at 292.9 eV and 295.7 eV, respectively, with an energy separation of 2.8 eV. The BE of potassium ions strongly depends on the degree of hydration, with increased concentration of water, i.e. the K 2p BE in KOH is known to be shifted to the lower energy^37^. The 2p_3/2_ spin–orbit for K_2_V_6_O_16_·2.7H_2_O is centered at 291.3 eV^27^, while for K_2_V_6_O_16_·1.5H_2_O is located at 292.6 eV^30^. The position of the K^+^ spin–orbit for both samples confirms the presence of fewer than 1.5 molecules of water in the structure, which is in good agreement with TG results. The survey spectra show that both samples consist only K, V, and O, and no other elements were detected (Fig. S5e,f, ESM). The analysis of the vanadium valence state in the bulk were characterized by the X-ray absorption spectroscopy. Figure 1e,f show the X-ray absorption near-edge structure (XANES) spectra of the V K-edge for KVO-20 and KVO-40, respectively. Both samples have the same pre-edge adsorption peak position, which is attributed to the forbidden electronic transition 1 s → 3d^42^. The vanadium K-edge position for vanadium oxides (V_2_O_3_, V_2_O_4_, and V_2_O_5_) show near to linear relation between the V oxidation state and the edge position (see insets on Fig. 1e,f). With for higher oxidation state the edge position shifts to a higher energy. The relative contributions in a mixed system can be estimated by performing a fit with a linear combination of known references. The determination of the oxidation state typically refers to a shift of the absorption edge^43^. To determine the “edge position”, the maximum of the first derivative of the XANES spectrum was used^44^. The maximum of the first derivative of the KVO-20 and KVO-40 XANES spectra were observed near to the maximum of the V_2_O_5_ reference (Fig. 1). This suggest that the valance state of vanadium is 5+. It should be noticed that the transmission mode XAS analysis gives information about the bulk, while the XPS is surface-sensitive method, which explain the differences in the obtain results. It is commonly reported that the amount of V^4+^ can reach up to 10% of the total amount of vanadium ions on the surface, especially when the synthesis is performed by wet-chemical method^6^. Summarizing, the chemical formula of the KVO samples based on MP-AES, XPS, XAS and TGA results is for KVO-20: K_2.44_V_6_O_16_·0.65H_2_O and for KVO-40: K_2.54_V_6_O_16_·0.76H_2_O.

**Figure 2:**
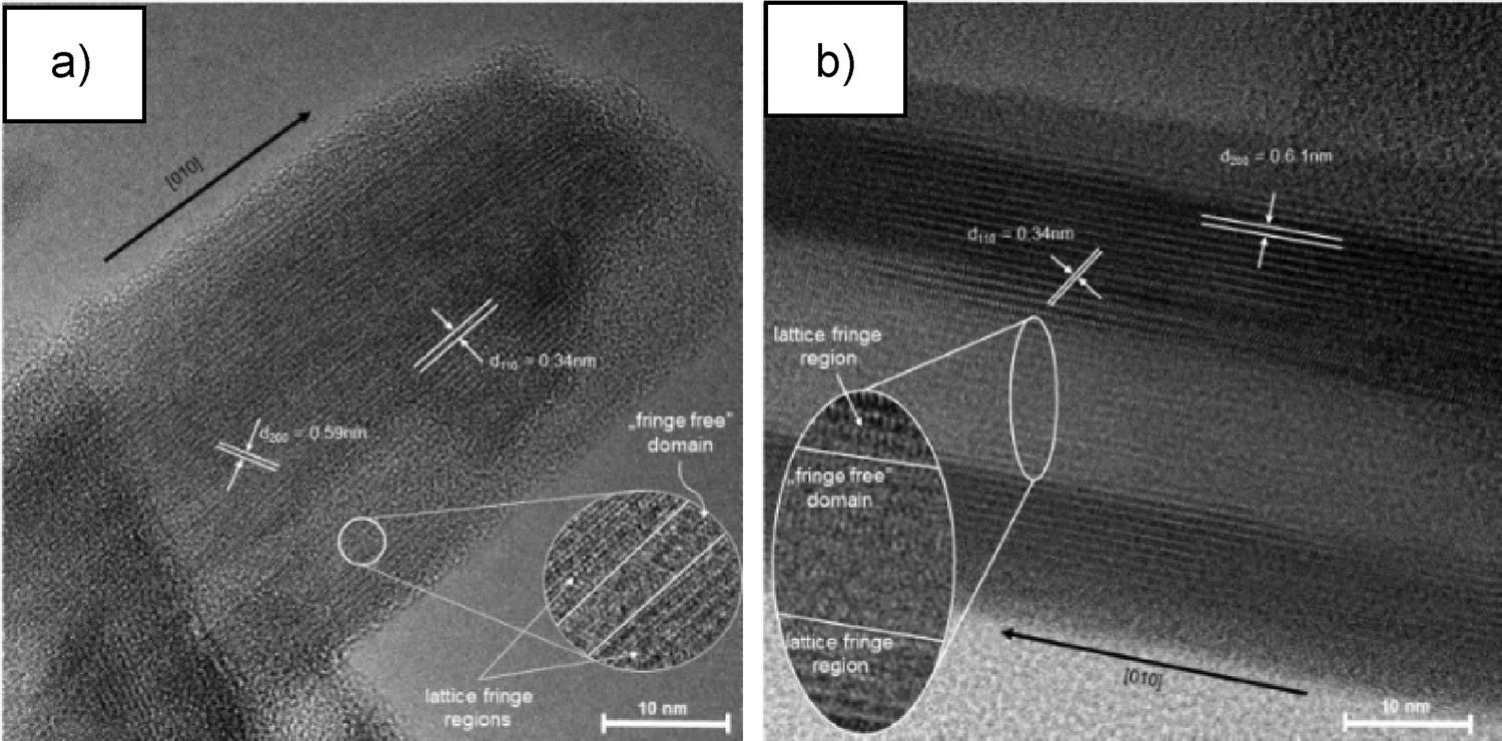
High-resolution TEM image of KVO-20 (**a**) and KVO-40 (**b**).

The sample KVO-20 shows a belt-like structure, where single nanobelts have a width 50–200 nm, a thickness of 10–40 nm, and a length of few micrometers (Fig. S6a). With increasing synthesis temperature, the nanobelts become thinner, and their cross-section decreases. The width of sample KVO-40 (Fig. S6b) nanostructures are between 30 and 50 nm, and the thickness is below 10 nm. Thinner and narrower belts result in a more developed surface area: the specific surface area was more than two times higher for KVO-40 (S_BET_ = 36.3 m^2^ g^−1^) than for KVO-20 (S_BET_ = 15.6 m^2^ g^−1^) (Fig. S6c). The TEM images of a single nanobelt of KVO-20 (Fig. 2a) and KVO-40 (Fig. 2b) reveal a region of “fringe-free” domains between clearly distinguishable lattice fringe regions. A similar structure with fringe-free domains was observed previously for Na_2_V_6_O_16_·3H_2_O nanorods^45^. This suggests that the nanobelts are composed of crystalline and amorphous domains arranged alternately. Moreover, the “fringe-free” domains were observed more often for KVO-20 than KVO-40. However, the hydrous layered potassium hexavanadates are unstable under the strong electron beam irradiation condition, leading to the destruction of their crystallines^29^. Therefore, to confirm that these are not artefacts, the picture was taken at the same position in the interval of about 10 s.

The regions with the fringe lattice and the “fringe-free” domain arranged alternately are present before and after irradiating with the electron beam (Fig. S7a,b, respectively, ESM). The formation of crystalline/amorphous structure is probably caused by the delamination process during sample synthesis. The structural defects present lead to structure collapse during this process, and the fringe-free domains creation. The concentration of fringe-free domains is dependent on synthesis temperature. At higher temperatures, it will be smaller because a more uniform crystal structure will be created. The observed lattice spacing for the crystalline region was calculated to be 0.34 nm and 0.59 nm for sample KVO-20 and 0.34 nm and 0.61 nm for sample KVO-40, which closely correspond to the (110) and (200) planes of the monoclinic K_2_V_6_O_16_·1.5H_2_O, respectively. Thus, the hydrated potassium vanadate nanobelts show a preferred [010] growth orientation direction (b-axis) and the width along the [100] direction (a-axis). The surface of the nanobelts is mainly formed by the (001) and (110) planes (Fig. S8b in the ESM). The structure of K_2_V_6_O_16_·1.5H_2_O (see Fig. S8a, ESM) consists of the infinite V–O chains along the b-axis with a short V–V distance of 0.18 nm. This is indicating the high-anisotropic internal structure with the highest stacking density along this direction. In comparison, the a-and c-axis have longer average V–V distances close to 0.30 and 1.12 nm, respectively and are characterized by a lower stacking density. Therefore, the growth rates for the a-and c-axis are slower than for the b-axis. As a result, the hydrated potassium vanadate nanobelts grow along the [010] direction. Additionally, along the c-axis, connected double chains have loosely-packed tunnel-interoperating structures, and considerably large interlayer distances form along the a-axis^46^.

### Electrochemical properties

**Figure 3:**
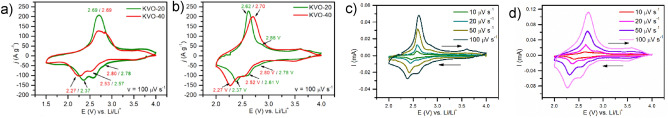
Cyclic voltammetry curves of KVO-20 and KVO-40 electrode materials at a scan rate of 100 µV s^−1^ over 1.5–4.0 V (**a**) and 2.0–4.0 V (**b**) potential window. Cyclic voltammetry curves at different scan rates for (**c**) KVO-20 and (**d**) KVO-40.

**Figure 4:**
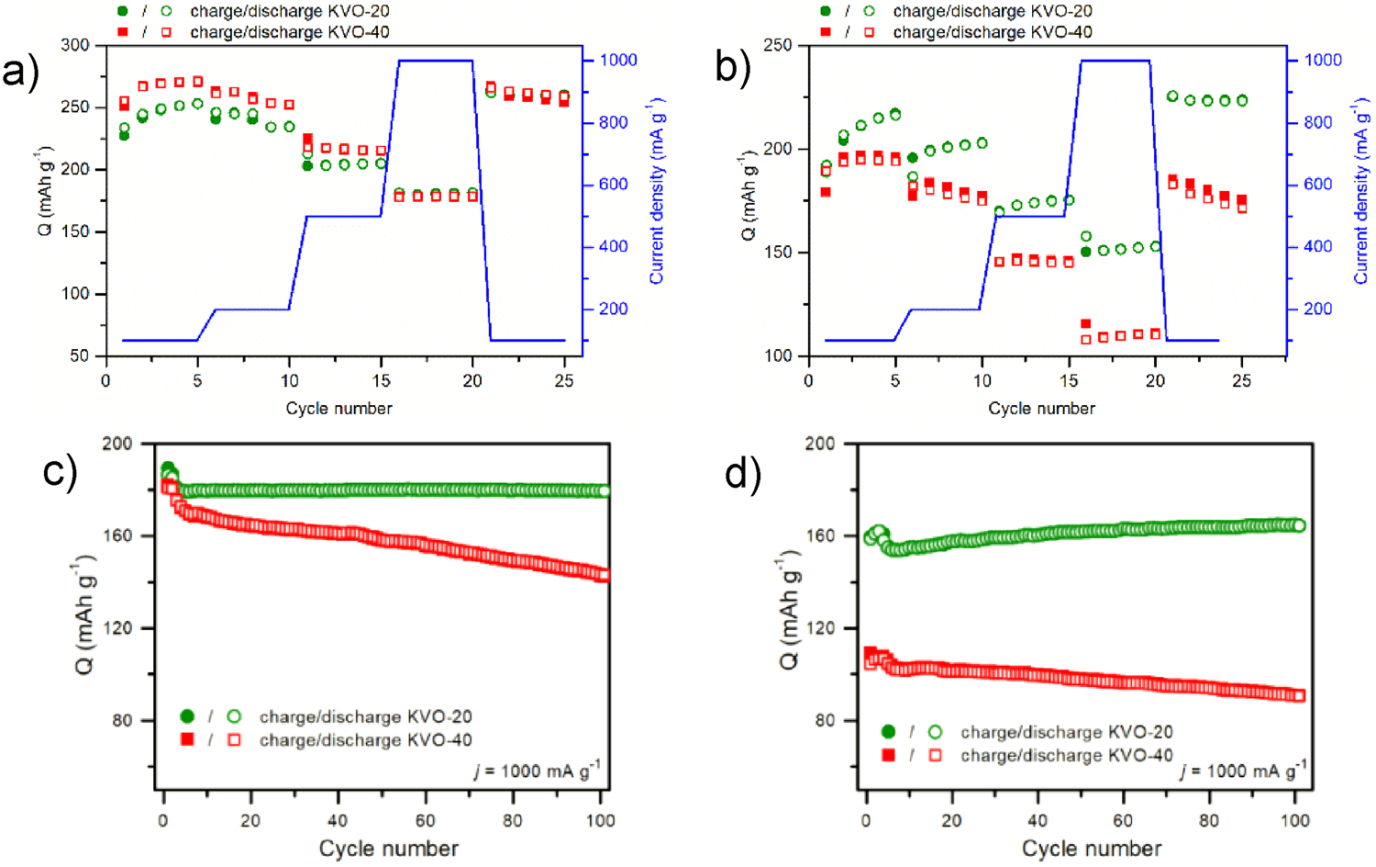
Electrochemical performance of KVO-20 and KVO-40 electrode materials: Galvanostatic tests over the voltage range of (**a**) 1.5–4.0 V and (**b**) 2.0–4.0 V. The charge/discharge tests at j = 1 A g^−1^ for 100 cycles in (**c**) 1.5-4 V and (**d**) 2.0–4 V.

Cyclic voltammetry of the KVO-20 and KVO-40 electrodes at a sweep rate of V = 100 µV s^−1^ is shown in Fig. 3a,b. One may see that during the reduction process, two current maxima are observed for both electrode materials. The position of those maxima depends on the type of electrode material: 2.37 V, 2.57 V (Fig. 3a) or 2.61 V (Fig. 3b) (with a shoulder at 2.78 V) for KVO-20 and 2.27 V, 2.53 V (Fig. 3a) or 2.52 V (Fig. 3b) (with a shoulder at 2.80 V) for KVO-40. The peaks located between 2.6 V and 2.8 V are attributed to the reduction of vanadium ions from V^5+^ to V^4+^. The current maximum at ~ 2.4 V is originating to vanadium V^5+^ to V^4+^ reduction and partial reduction of V^4+^ to V^3+^^20^. The former reaction is a dominant process. The shift in position is very likely to be attributed to the difference in the vanadium valence state of active material. The reduction of V^5+^ to V^4+^ occurs for the sample KVO-20 at the lower potentials since the concentration of V^4+^ ions in this sample is five times greater than for KVO-40. The presence of two well-defined current maxima is related to the insertion of two lithium ions atoms into the KVO-20 and KVO-40 layers. It is very interesting that during the oxidation process there is only one anodic maximum for both electrode materials. The anodic current maximum is at potential ca. 2.70 V. It might be attributed to the fact that during oxidation the lithium ions are removed from vanadium oxide layers simultaneously. To determine the charge storage mechanism the method proposed by Liu et al. was performed^47^. In general, two electrode processes occur during the examination of the sweep-rate dependence of the current response during cyclic voltammetry procedure: capacitive (a surface mechanism) and diffusion dependent. The total current response of an electrode is a mixture of these mechanisms:

1 $$i\left( {\text{V}} \right) \, = {\text{ k}}_{{1}} \cdot v + {\text{ k}}_{{2}} \cdot v^{{{1}/{2}}} ,$$

where k_1_·v is the capacitance related process while k_2_·v^1/2^ is attributed to diffusion-controlled mechanism. One should take into account that in a real system simple mechanism does not occur^48^. To evaluate the charge storage contribution the analysis of the sweep-rate of voltammetric currents was performed, see Fig. 3c,d. Detailed analysis is presented in Fig. S9. The shaded regions show the percentage contribution of capacitive currents in the total charge storage. It is seen that even for low sweep rates the charge storage is mainly attributed to the surface mechanism, and equal to about 80%. We also performed the GITT measurements to calculate diffusion coefficient of KVO-20 and KVO-40 electrode materials (Fig. S10). For KVO-20 (Fig. S10a, ESM) and KVO-40 (Fig. S10b, ESM) during reduction process the value of diffusion coefficient continuously increases. During oxidation, the increase is seen for KVO-20 in the full range while for the KVO-40 only till 2.6 V followed gradual decrease with the increase of the voltage. There is a visible relation between the values of diffusion coefficient and redox couple activity of electrode materials. The current responses related to redox/oxidation couple activity is in the potential range from around 2.1 to 2.8 V. There is an increase in the diffusion coefficient within this range for both processes. It shows that the structure of KVO seems to be flexible for lithium insertion/extraction. Such flexibility may be attributed to inclination of potassium ions to be replaced by lithium ions to obtain an equilibrium. The KVO-20 and KVO-40 materials were tested over two potential ranges 1.5–4.0 V and 2.0–4.0 V, at current density ranging from 100 to 1000 mA g^−1^. The relation between specific capacity and applied current density is given in Fig. 4a,b). One may see that during the first five cycles at current density *j* = 100 mA g^−1^ for both electrode materials the value of specific capacity slightly increases. Comparing the specific capacity of electrode materials charged/discharged in the two potential ranges, obtained values are about 50 mAh g^−1^ higher for 1.5–4.0 V in than for the 2.0–4.0 V. The specific discharge capacity of KVO-20 and KVO-40 electrode material for *j* = 1 A g^−1^ is 181 mAh g^−1^ for both materials investigated in the potential range from 1.5 to 4.0 V (Fig. 4a). The obtained value is higher than specific discharge capacity reported recently for similar vanadium oxide- based materials such as Na_2_V_6_O_16_·xH_2_O nanowires^49,50^, and CaV_6_O_16_.3H_2_O nanoribbons^51^. In the narrower potential range, the specific discharge capacity was equal to 153 mAh g^−1^ and 110 mAh g^−1^ for KVO-20 and KVO-40, respectively (Fig. 4b). It is noteworthy that there are no significant differences in specific capacities for KVO-20 and KVO-40 electrode material polarized in 1.5–4.0 V potential range for different current densities. Moreover, after applying the current density of 1 A g^−1^, both electrode materials showed similar remarkable specific capacity values for the current density of 100 mA g^−1^ (cycles from 21 to 25) equal to 260 mAh g^−1^.

Unfortunately, the KVO-40 electrode material exhibited much worse electrode performance for the potential range 2.0–4.0 V. It is In analogy to similar described compounds i.e. Na_2+x_V_6_O_16_ system^52^, it is possible that only up to 2 lithium ions can be up taken by KVO. During intercalation lithium ions move into the structure according to reactions:

2 $${\text{K}}_{2.44} {\text{V}}_{6} {\text{O}}_{16} \cdot 0.65{\text{H}}_{2} O \, + {\text{ Li}}^{ + } + \, 2e^{ - } = {\text{ Li}}_{2} {\text{K}}_{2.44} {\text{V}}_{6} {\text{O}}_{16} \cdot 0.65{\text{H}}_{2} {\text{O for KVO}} - 20,$$

3 $${\text{K}}_{2.54} {\text{V}}_{6} {\text{O}}_{16} \cdot 0.76{\text{H}}_{2} {\text{O}} + {\text{ Li}}^{ + } + \, 2e^{ - } = {\text{ Li}}_{2} {\text{K}}_{2.54} {\text{V}}_{6} {\text{O}}_{16} \cdot 0.76{\text{H}}_{2} {\text{O for KVO}} - 40.$$

Consequently, the theoretical capacity is 80 mAhg^−1^ and 79 mAhg^−1^ for KVO-20 and KVO-40, respectively. In the studied case the specific capacity for both cathode materials is much higher than the theoretical value. Thus, the comparison of theoretical capacity with obtained capacity is misleading. The theoretical capacity refers to insertion of lithium ions while the total capacity is a sum of capacities originating from intercalation (diffusion of lithium ions into the material structure) and charge storage on the surface of the material (pseudofaradaic). The obtained results evidenced that a surface mechanism is a dominant process, and is equal to about 80%, see Fig. S9 in the ESM.

**Figure 5:**
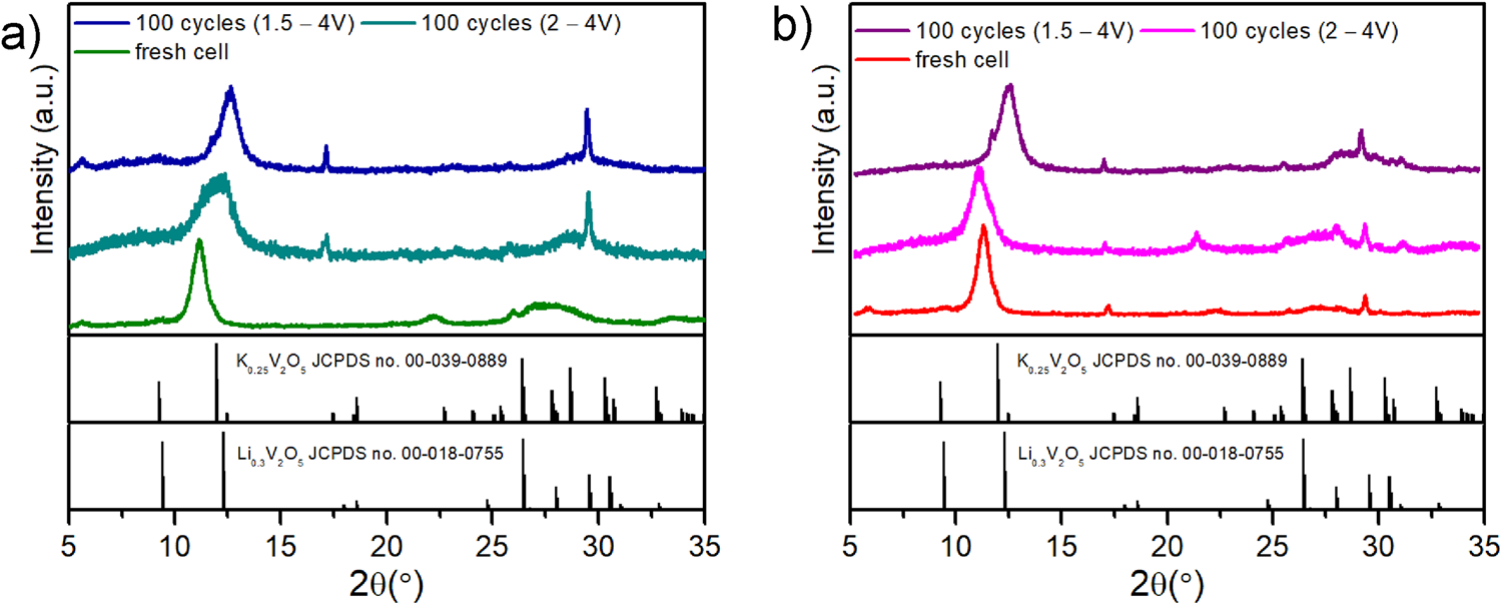
Ex-situ XRD pattern of the (**a**) KVO-20 and (**b**) KVO-40 electrode at the pristine state and a fully discharged state electrode after 100 charges/discharge test at j = 1 Ag^−1^ in two potential ranges.

The charge storage mechanism by KVO system is pseudofaradaic origin where the charge is stored via redox reaction mainly at the surface^53^ and not into the structure of the material. The charge storage contribution is almost the same for KVO-20 and KVO-40 electrode materials. It evidences that in terms of chemical formula both materials are similar. Moreover, the differences in specific surface area (15.6 m^2^ g^−1^, and 36.3 m^2^ g^−1^ for KVO-20 and KVO-40, respectively) do not influence the charge storage mechanism. Thus, the obtained differences of the specific capacity values are rather due to the differences in the crystallographic structure than in the difference in chemical composition. In other words, the slight differences in chemical composition affect the crystallographic structure that has a huge impact on electrochemical performance. Such difference might likely be due to the differences in the initial vanadium valence state on the sample surface of KVO-20 and KVO-40 samples. It was reported that the valence state of vanadium plays a crucial role in the electrochemical performance of vanadium oxide as cathode materials^31^. The presence of V^4+^ is beneficial for ion insertion/extraction, as that the presence of an appropriate number of tetravalent vanadium would is beneficial for the electronic transfer^54^. Considering the high values of the specific capacity at 1 A g^−1^, extended cycles at a given current density were performed to investigate the cycling performance (Fig. 4c,d).The electrochemical behavior of KVO-20 and KVO-40 electrode materials for 100 charge/discharge cycles suggests that charge storage might be of capacitive origin in both cases. The KVO-20 electrode material seems to be more stable during electrochemical tests in both potential ranges and additionally exhibit higher specific capacity values. The 5th cycle discharge capacity in the 1.5–4.0 V potential range is 179 mAh g^−1^, and the capacity retention is 99.89% for the 101st cycle (Fig. 4c). In the narrower potential range, the specific capacity shows an increasing trend between the 5th and 101st cycle, from 154 to 164 mAh g^−1^. This phenomenon might be attributed to structural changes in the sample during cycling. In the case of KVO-40 electrode material for the 1.5–4.0 V potential range, the specific capacity equals 170 mAh g^−1^ for the 5th discharge cycles and the 101st cycle drops to 143 mAh g^−1^ with the capacity retention of about 85% (Fig. 4d). In the narrower potential range, where the initial discharge capacity is 106 mAh g^−1^, the capacity retention between the 5th and 101st discharge cycle is similar and equals 83%. Intercalation of two Li ions into vanadium compounds, results in a series of crystalline-to-crystalline transitions. The intercalation of the first Li-ion, brings only minor structural changes, while the insertion of the second Li-ion leads to significantly different structures, often irreversible^55^. We assume that such process may led to off-stoichiometric defects that affects lithium intercalation/extraction process and specific capacity of the material. Similar phenomenon was previously observed for LiFePO_4_ by Ceder et al.^56^. They noticed that cycling at higher current density affects the specific capacity of studied electrode materials. The presence of plateau on both galvanostatic profiles is typical for the faradaic reaction taking place in both systems even after cycles (Fig. S12). The position of the plateau agrees with the current maxima observed in cyclic voltammetry curves (Fig. 3a,b). Nevertheless, the investigation of the structural changes of cathode material after cycling with the utilization of the ex-situ XPS, XAS, XRD, MP-AES were performed. The electrodes were examined at a fully discharged (oxidized) state. Figure 5 shows the XRD pattern of fresh electrode material and after 100 galvanostatic tests cycles in both potential ranges. The structure of the KVO-20 and KVO-40 samples did not preserve pristine crystal structures after cycling. However, the clear diffraction peaks are visible (Fig. S13), which confirms the preservation of the crystalline structure of the samples after charge/discharge cycles. After 100 cycles in the 2.0–4.0 V range, the (002) peak was shifted from 11.15° to 12.10° for sample KVO-20 and 10.90° for sample KVO-40. The shift of the (002) peak position to lower angle values for KVO-40 electrode indicated that during uptake/removal of Li^+^ from KVO-40 lattice, a small amount of Li^+^ might have remained trapped in the crystal structure^54^. For the broader potential range, the (002) Bragg peak was shifted from 11.15° to higher angles for both samples. For the sample KVO-20 the peak is located at 12.54° after 100 cycles. Whereas, for the sample KVO-40, the (002) Bragg peak is shifted to 12.46° after 100 cycles.

**Figure 6:**
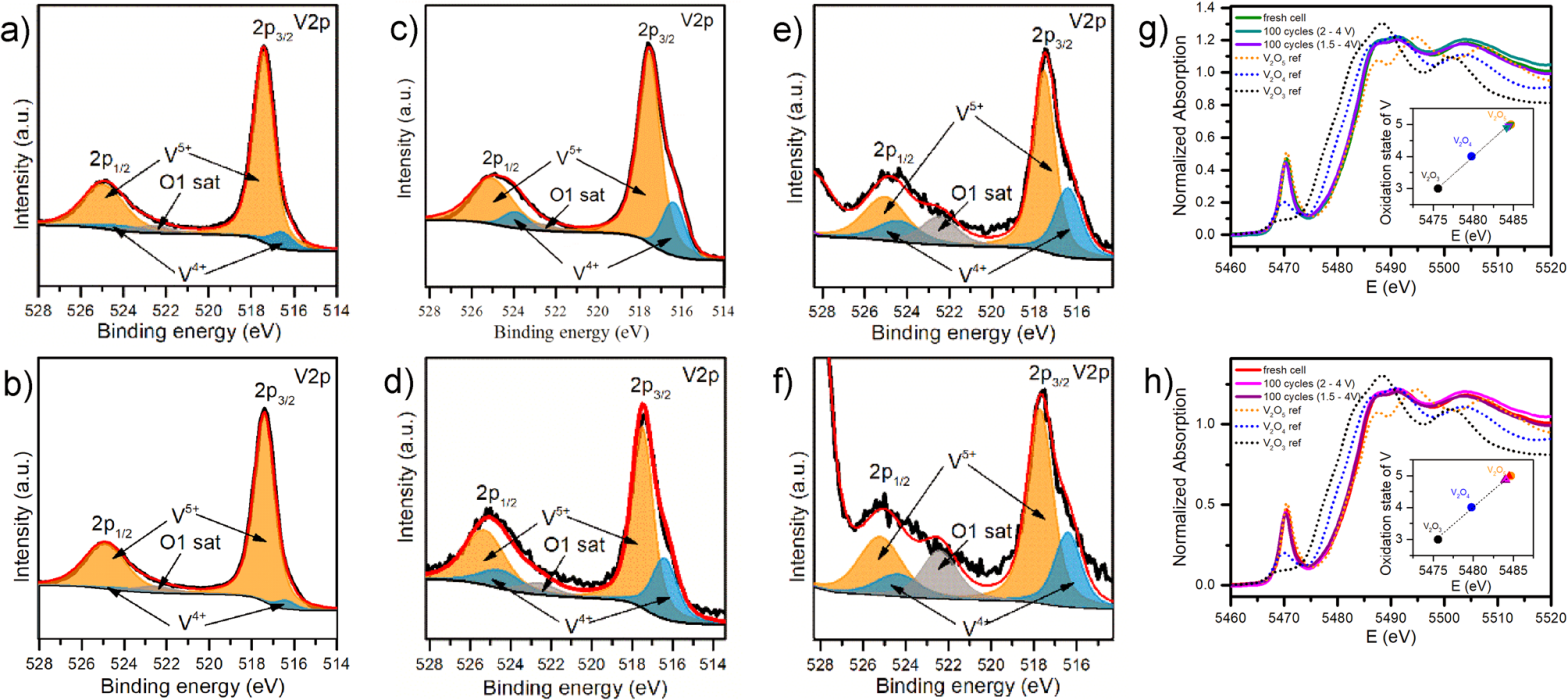
(**a–f**) *Ex-situ* high resolution XPS spectra of the V2p regions in pristine (**a**) KVO-20, and (**b**) KVO-40 electrode, and a fully discharged state of the KVO-20 and KVO-40 electrode after 100 charges/discharge test at j = 1 A g^−1^ in two potential ranges: 2.0-4 V (**c, d**) for KVO-20 and KVO-40 respectively), and 1.5–4 V (**e,f**) for KVO-20 and KVO-40 respectively); (**g,h**) Ex-situ V K-edge XANES spectra of the pristine electrodes and a fully discharged state after 100 charges/discharges test at j = A g^−1^ in two potential ranges: 2.0–4.0 and 1.5–4.0 for (**g**) KVO-20 and (**h**) KVO-40, respectively.

The ex-situ XPS analysis (Fig. 6) showed that the ratio of V^4+^/V^5+^is increasing after cycling in comparison to the initial samples (Table S2). The significant change of V^4+^ concentration, over 20 times greater, is observed for KVO-40 material after 100 cycles in 2.0–4.0 V potential range, whereas for KVO-20 material, the V^4+^/V^5+^ ratio is doubled. After charge/discharge cycles in broader potential range, the V^4+^/V^5+^ ratio is the same for both samples, and equal to 40 after 100 cycles. The ex-situ V K-edge XANES analysis (Fig. 6) showed that the vanadium oxidation state of pristine electrodes is the same as for the powdered sample and is equal to 5+. After 100 charged/discharged cycles in both potential ranges, the average oxidation state of V is lower than for V_2_O_5_. Based on the maximum of the first derivative of the XANES spectrum, the mixed valence state of vanadium for electrodes after cycling was estimated. After 100 cycles in the 2.0–4.0 V potential range, the valence state of vanadium in the KVO-20 material (Fig. 6g) is 4.95, where after cycling in wider potential range equal to 4.90. Thus, the V^4+^ concentration equals 5% and 10%, respectively. Whereas, for the KVO-40 material (Fig. 6h) after 100 cycles in both potential ranges, the vanadium oxidation state is around 4.9, it suggests that about 10% of V^4+^ ions are in the bulk KVO-40 material. The shift of the (002) Bragg peak evidenced that stoichiometry of fresh material undergoes structural changes due to this replacement. To confirm this phenomenon, the MP-AES measurements (microwave plasma-atomic emission spectroscopy) were performed. The MP-AES analysis confirmed that the relative atomic ratio of V/K in both samples is increasing after galvanostatic cycles, and depends on the potential range and number of cycles (Table S2). Moreover, the presence of the lithium was detected, for all samples. The relative atomic ratio of V/Li is close to one after cycles in broader potential range, whereas for narrower potential range is almost 5-times greater The XRD pattern of KVO-20 after CD cycles in narrower potential range agrees with two phases: K_0.25_V_2_O_5_ (JCPDS no. 00-039-0889) and Li_0.30_V_2_O_5_ (JCPDS no. 00-018-0755). This indicated that potassium was gradually removed from the crystal structure in the subsequent cycles and replaced with lithium, and probably intermediated phase (Li_x_K_y_V_6_O_16_) is created. In K_0.25_V_2_O_5_ the potassium is known to act as a “pillar” connecting the adjacent V–O layers and leading to more stable interlayer structure as well as preventing the relative slippage of two adjacent V–O layers^12,22,23^.

We believe that this flexibility of the KVO-20 structure was caused by the higher contribution of V^4+^ ions on the sample surface, which could facilitate the potassium removal from the lattice. This phenomenon was probably related to the observation of increasing specific capacity with cycles, see Fig. 4a,b. The MP-AES analysis (Table S3, ESM) confirmed that the relative atomic ratio of V/K changed from 3.9 (pristine electrode) to 6.89 (potential range 1.5–4 V, Fig. 4a) and to 5.38 (potential range 1.5–4 V, Fig. 4b). Moreover, the atomic ratio of V/Li equals 1.40 and 4.70 for sample KVO-20 after 100 cycling in the 1.5–4.0 V and 2.0–4.0 V potentials range, respectively. These results suggest that structural changes of the KVO-20 sample occur faster in the 1.5–4.0 V potential range, and the phase with higher capacity formed at the beginning of the cycling. Therefore, we do not observe a gradual increase of specific capacity (Fig. 4a). In the case of the narrower potential range, the structural changes occurred gradually (Li_x_K_y_V_6_O_16_, with continuous increase of x and decrease of y, cycle per cycle). Interestingly, the positions of indexed reflections on the XRD pattern suggested that after cycling, the KVO-40 material in 2.0–4.0 V potential range preserved the pristine structure, but the relative amount of potassium was smaller in comparison with no cycled material, and the presence of lithium in the structure was detected. Additionally, based on the XPS and XAS data, the contribution of V^4+^ was more dominant in the KVO-40 material after cycling. It was likely related to the fact that the higher capacity fading was observed for KVO-40 material in comparison with KVO-20. The lithium concentration significantly increased when KVO-40 material cycled in both potential ranges were compared. The XRD patterns of KVO-40 after galvanostatic cycles in broader potential range corresponded to the Li_0.30_V_2_O_5_ (JCPDS no. 00-018-0755). This suggests that in the 1.5–4.0 V potential range, the K_2_V_6_O_16_·nH_2_O structure is more flexible. However, the MP-AES results showed that the K to Li exchange occurs faster for KVO-20 material than for KVO-40 material. Therefore, after reaching ~ 100 cycle in wider potential range, KVO-20 material exhibits more gradual capacity drop than KVO-40 material (Fig. 4c), because the irreversible structural changes occur faster. We assumed that the initial V^4+^/V^5+^ ratio has an impact on these processes. The KVO-20 sample has a 5-times greater concentration of V^4+^ on the surface than for sample KVO-40. We believe that this initial V^4+^ concentration is crucial to structural changes during cycling. Moreover, the structural changes in the sample KVO-40 lead to a faster increase of V^4+^ vacancies, both on the surface and in the bulk, which relates to constant structure damages, and the capacity is constantly declining. In the case of sample KVO-20, the concentration of V^4+^ is changing gradually. Moreover, the greater quantity of fringe-free domains in the KVO-20 sample prevents the structure from collapsing during replacing potassium with lithium within subsequent charges/discharges cycles. The comparison of electrochemical performance of the KVO-20 and KVO-40 electrode material with potassium vanadium compounds for LiBs is given in Table S4.

## Conclusions

In summary, the single-phase K_2_V_6_O_16_·nH_2_O nanostructures were successfully synthesized by the novel LPE-IonEx method. Obtained samples have the same structure, comparable water content, similar shape, and size but they vary with V^4+^ contribution. For the first time, the Li-ion storage performance of layered hydrated potassium hexavanadate nanobelts cathode was reported, which delivered a remarkable capacity of 260 mAh g^−1^ at 100 mA g^−1^ and good cycling performance with a high capacity retention of 99.98% after 100 cycles at 1 A g^−1^. The electrochemical performance, as well as the structural flexibility of K_2_V_6_O_16_·nH_2_O, strongly depends on the vanadium valence state. The charge on hydrated potassium vanadate is stored via redox reaction mainly at the surface. Thus, via the presence of the V^4+^ on the surface, the electronic transfer is facilitated, and higher capacity is achievement. Moreover, the higher V^4+^ surface concertation leads to faster structural changes during subsequent charge/discharge cycles. The ex-situ characterization by XRD and MP-AES shows that during the subsequent charge/discharge cycle, the potassium ions in the K_2_V_6_O_16_·nH_2_O structure are replacing by lithium. However, the higher initial concentration of V^4+^ leads to increased vacancies gradually during cycling, both on the surface and in the bulk as confirmed by the ex-situ XPS and XANES analysis. Therefore, the structural damage occur slowly. The unusual construction of the nanobelts, composed of crystalline and amorphous domains arranged alternately, probably also prevents the crystal structure from collapsing during this exchange. The obtained K_2_V_6_O_16_⋅nH_2_O cathode materials with unique morphology and structure might open a new approach for rechargeable multivalent metal ions in lithium-ion batteries.

## Supplementary Information

Supplementary Information.

## Data Availability

The datasets used and/or analysed during the current study available from the corresponding author on reasonable request.
