# Supplementary material for: The valance state of vanadium-key factor in the flexibility of potassium vanadates structure as cathode materials in Li-ion batteries
Source: Sci Rep. 2022 Nov 5;12:18751. doi: 10.1038/s41598-022-23509-x (PMC9637123; doi:10.1038/s41598-022-23509-x)
Supplement: Supplementary file 1 — Supplementary Information. [file 41598_2022_23509_MOESM1_ESM.docx]

Supporting Information

**The valance state of vanadium- key factor in the flexibility of potassium vanadates structure as cathode materials in li-ion batteries**

Marta Prześniak-Welenc^*^, Małgorzata Nadolska, Kacper Jurak ,Jinjin Li, Karolina Górnicka, Aleksandra Mielewczyk-Gryń, Małgorzata Rutkowska, Aandrzej Paweł Nowak





**Figure S1**. XRD pattern of KVO-20 and KVO-40.


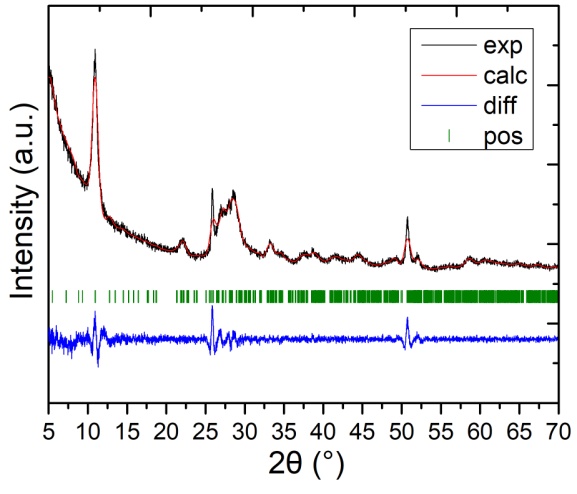




a)

b)

**Figure S2.** Le Bail refinement of K_2_V_6_O_16_∙1.5H_2_O for KVO-20 (a) and KVO-40 (b). The experimentally obtained data are shown with a black line, calculated pattern is shown with a red line and the green vertical marks indicate expected Bragg reflections for K_2_V_6_O_16_∙1.5H_2_O. The blue line at the bottom shows the difference between the observed and calculated data.

**Table S1.** The lattice parameters obtained from Le Bail refinement

|  | | a | b | c | α | β | γ |
| --- | --- | --- | --- | --- | --- | --- | --- |
| KVO-20 | 12,23 | 3,58 | 16,21 | 90 | 93,33 | 90 |  |
| KVO-40 | 12,25 | 3,60 | 15,95 | 90 | 93,74 | 90 |  |





**Figure S3**. TG curves of KVO-20 and KVO-40


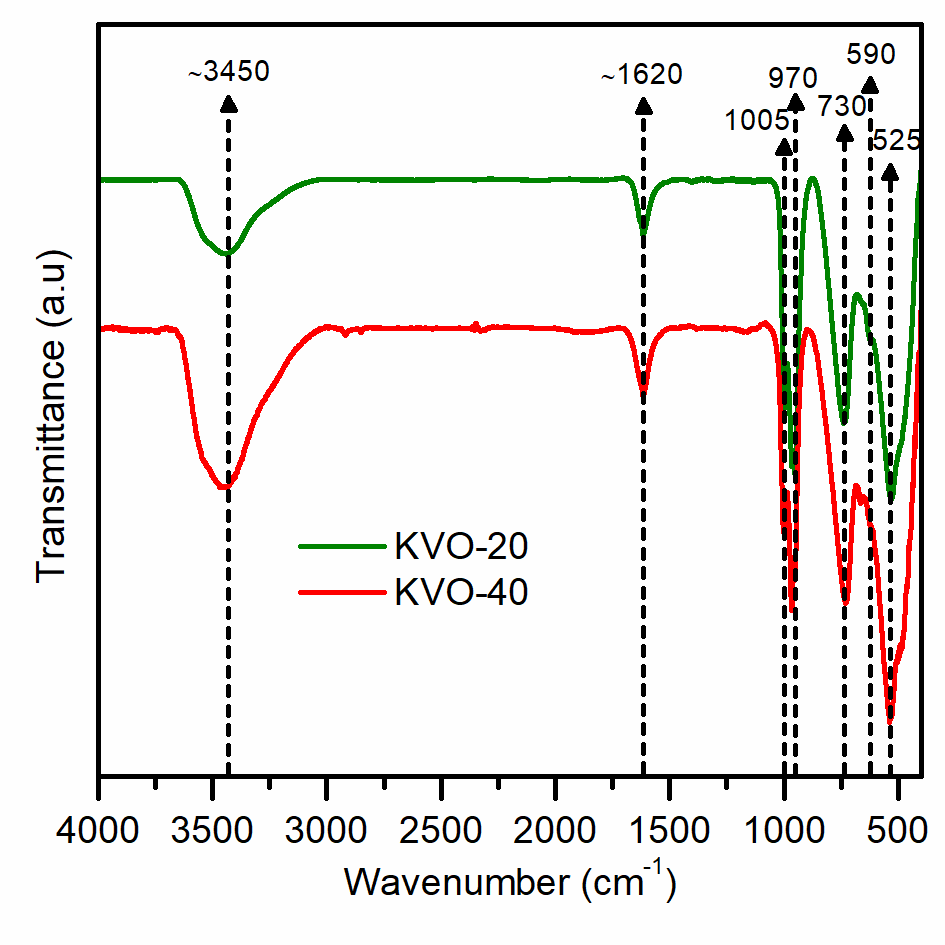


**Figure S4**. FTIR spectra of sample KVO-20 and KVO-40.

|  |   b) |
| --- | --- |
|   c) |   d) |
| 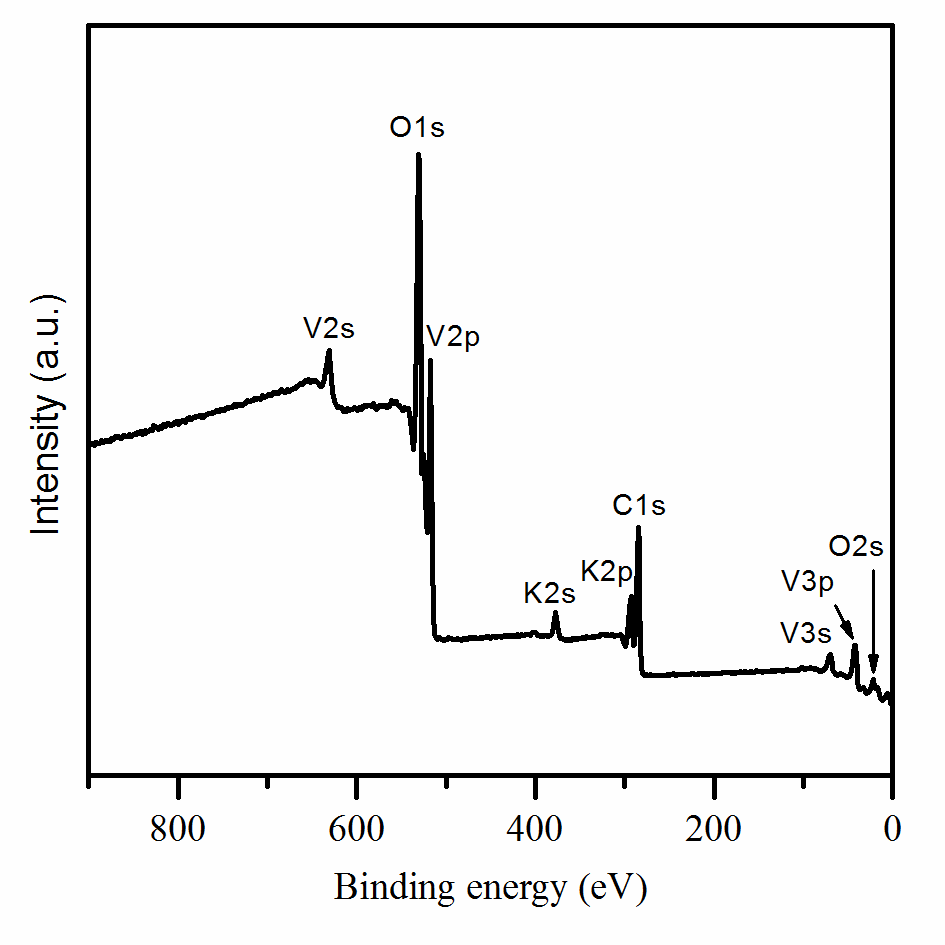  e) | 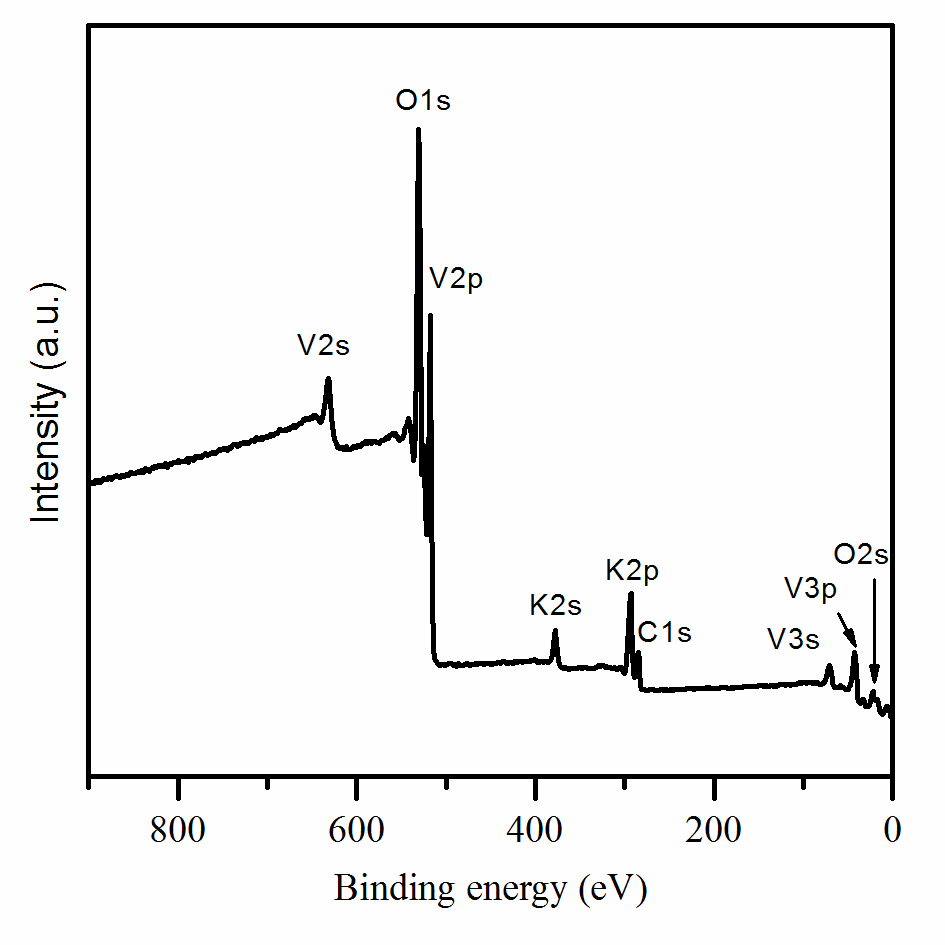  f) |

**Figure S5**. The XPS spectra of samples, (a) and (b) the 2p_1/2_ V2p region of KVO-20 and KVO-40 respectively, (c) and (d) K 2p spectrum of KVO-20 and KVO-40 respectively, (e) and (f) Survey spectrum of KVO-20 and KVO-40 respectively.

c)

b)

a)

| 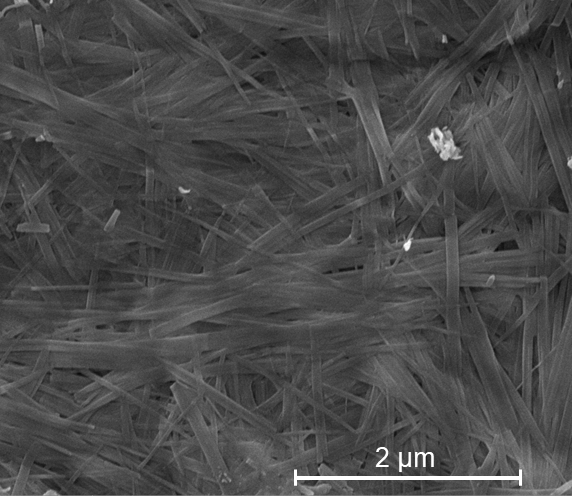  a) | 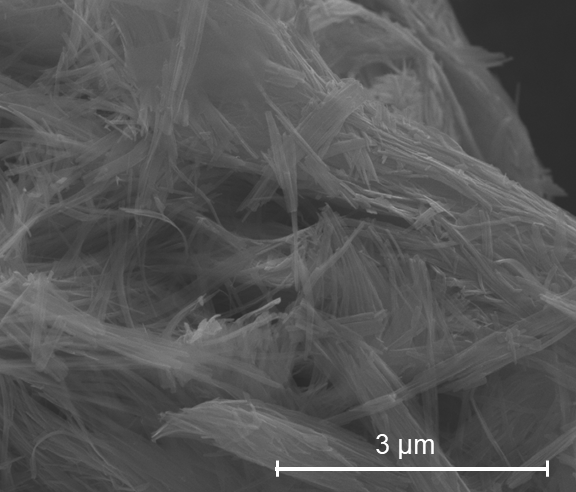  b) |
| --- | --- |


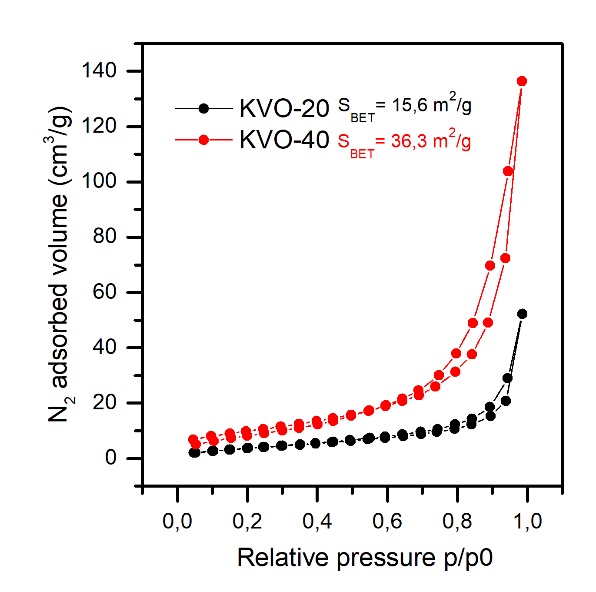


c)

**Figure S6.** a-b SEM images of KVO-20 (a) and KVO-40 (b), c N_2_ adsorption-desorption isotherm of KVO-20 (black) and KVO-40 (red) with calculated specific surface areas.

**a)**

**b)**


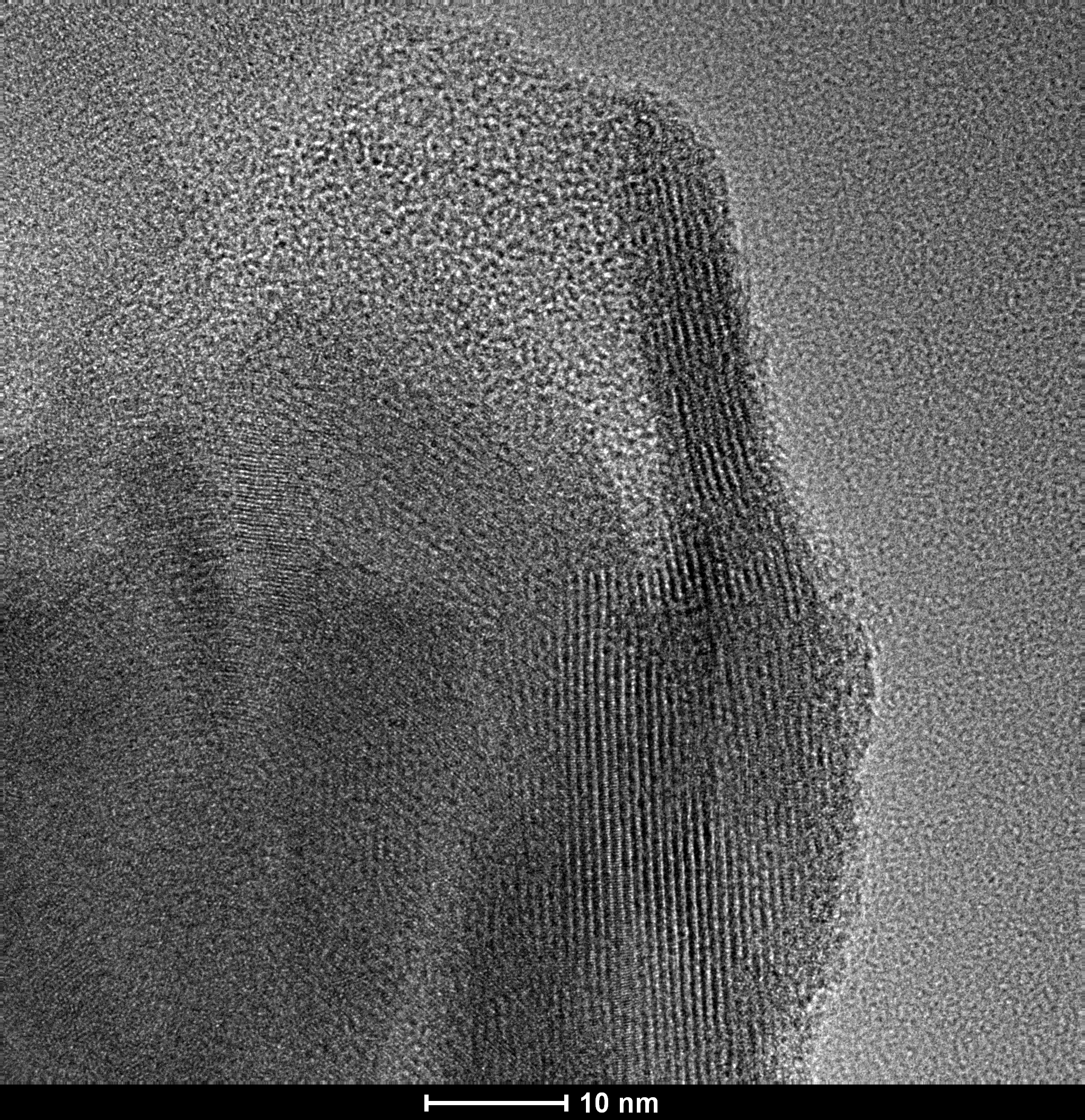

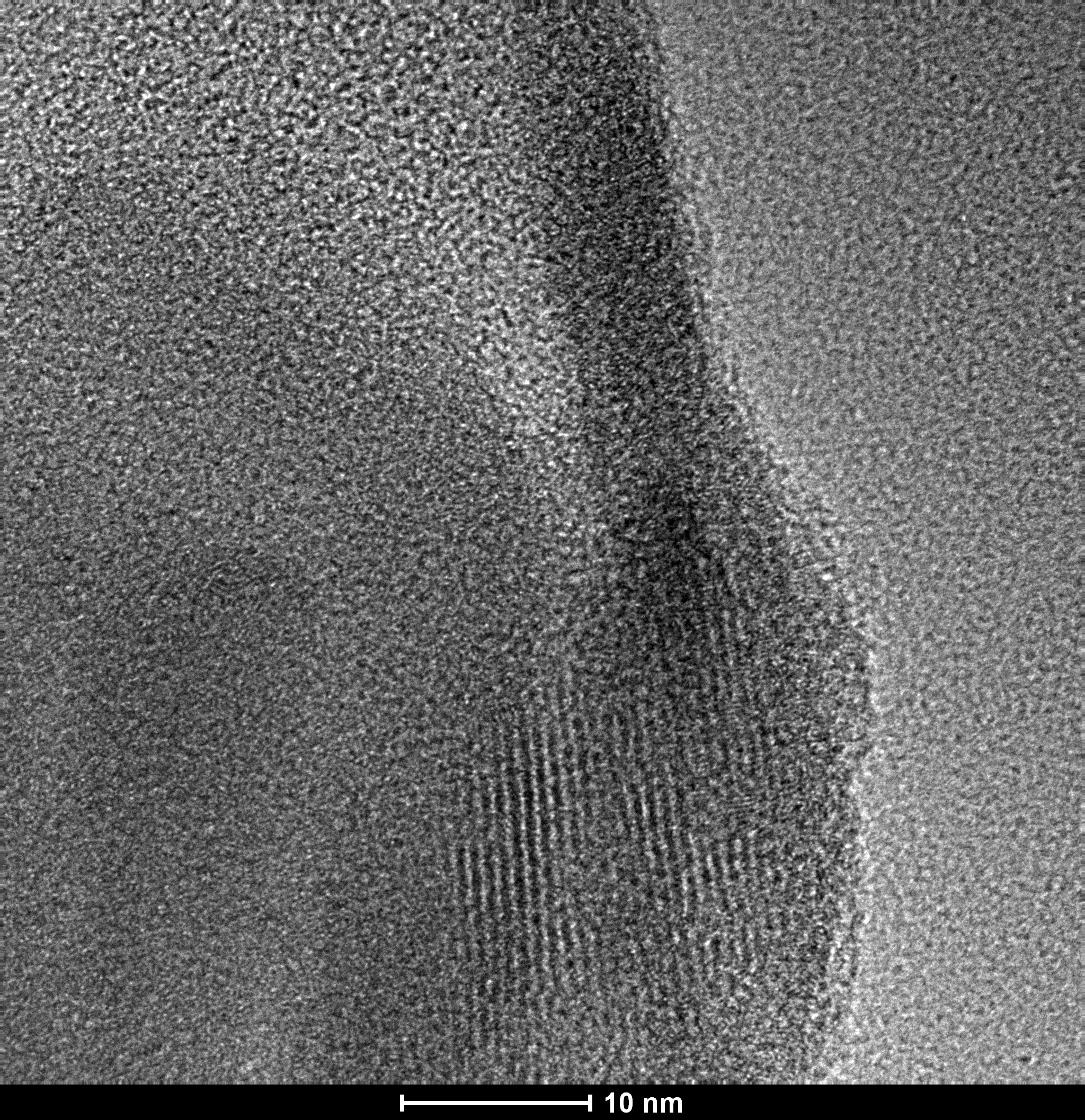

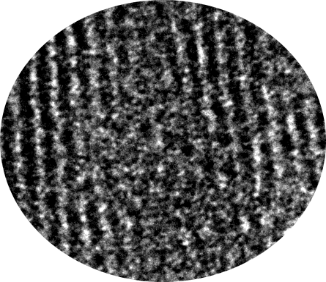

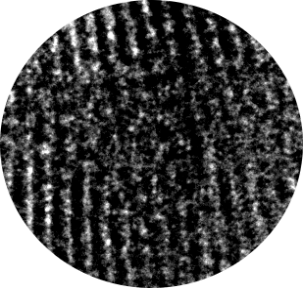


crystalline

phase

amorphous

phase

fringefree

domain

fringefree

domain

**Figure S7.** The HRTEM image of KVO-40 (a) of the same position as (b) after irradiating with the electron beam for 30 seconds


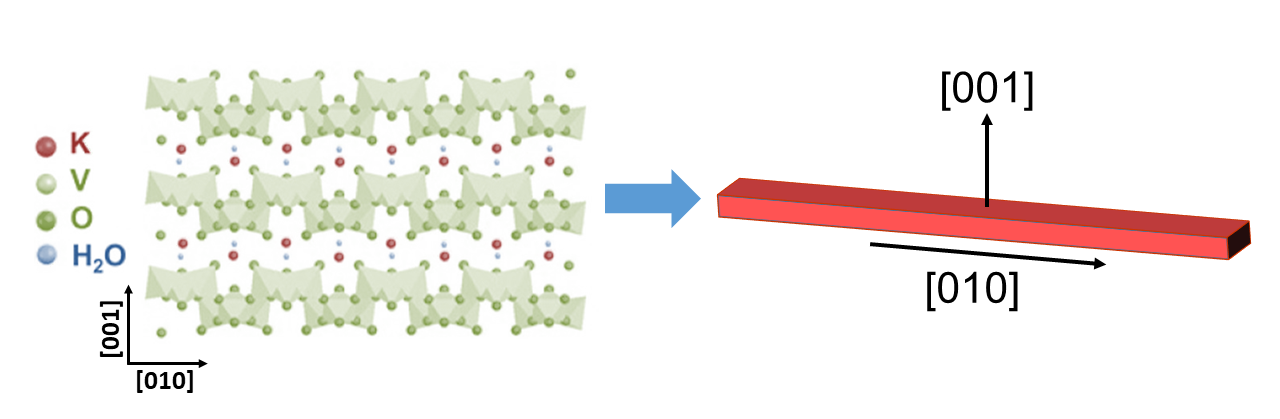


**Figure S8**. (a) Projection of the structure of K_2_V_6_O_16_∙1.5H_2_O along [010], (b) The schematic image of K_2_V_6_O_16_·nH_2_O nanobelt.

| 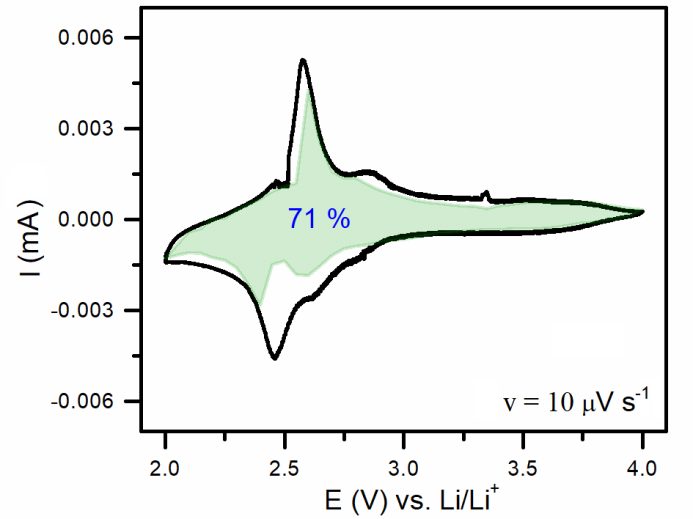  a) |   b) |
| --- | --- |
| 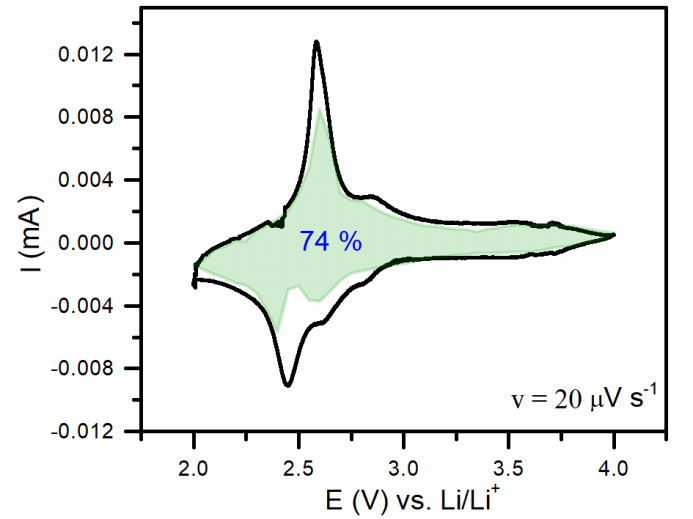  c) |   d) |
| 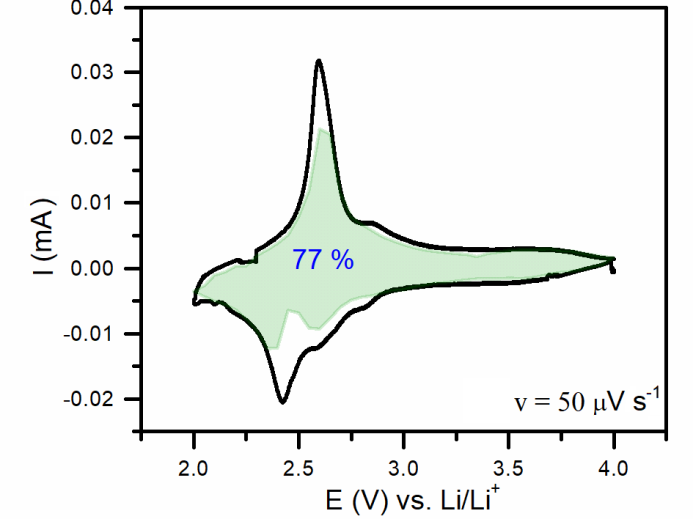  e) |   f) |
| 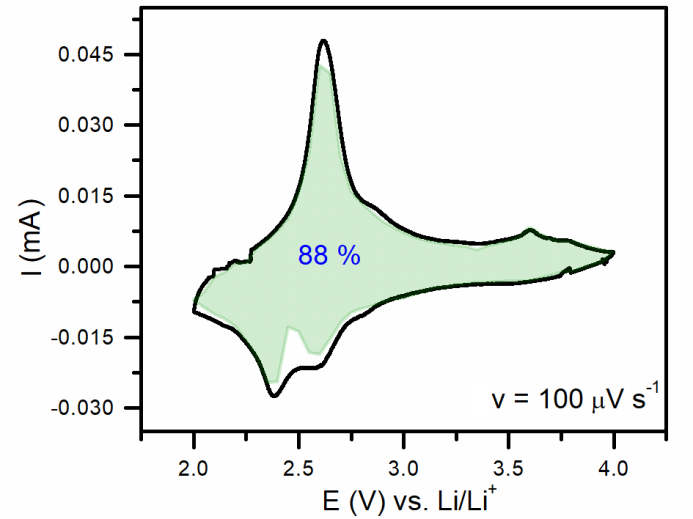  g) |   h) |

**Figure S9.** Charge storage contributions at (a) 10 μV s^-1^, (c) 20 μV s^-1^, (e) 50 μV s^-1^ and (g) 100 μV s^-1^ of KVO-20 electrode material and at (b) 10 μV s^-1^, (d) 20 μV s^-1^, (f) 50 μV s^-1^ and (h) 100 μV s^-1^ of KVO-40 electrode material. Shaded regions presents the capacitive currents with their percentage contribution in the total charge storage.

|   a) |   b) |
| --- | --- |

**Figure S10**. The calculated diffusion coefficient of KVO-20 (a) and KVO-40 (b) electrode material at the first cycle.





a)




**Figure S11**. The coulombic efficiency and capacity fade for a) KVO-20 and b) KVO-40 electrode material during 500 subsequent charge/discharge test at j = 1 A g^-1^ in 1.5-4V.

b)

| **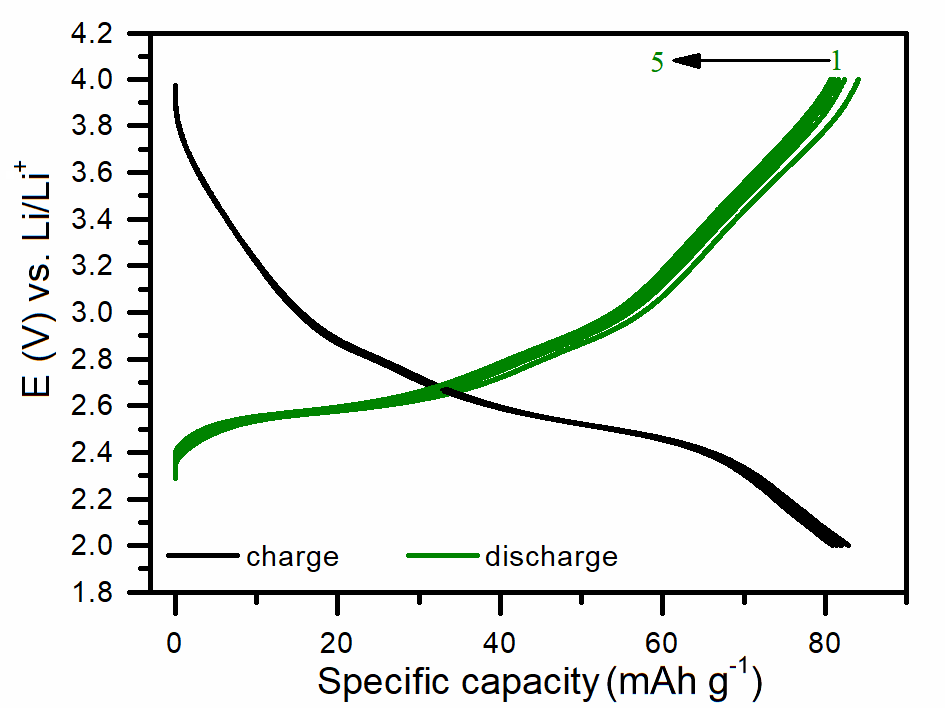**  a) | **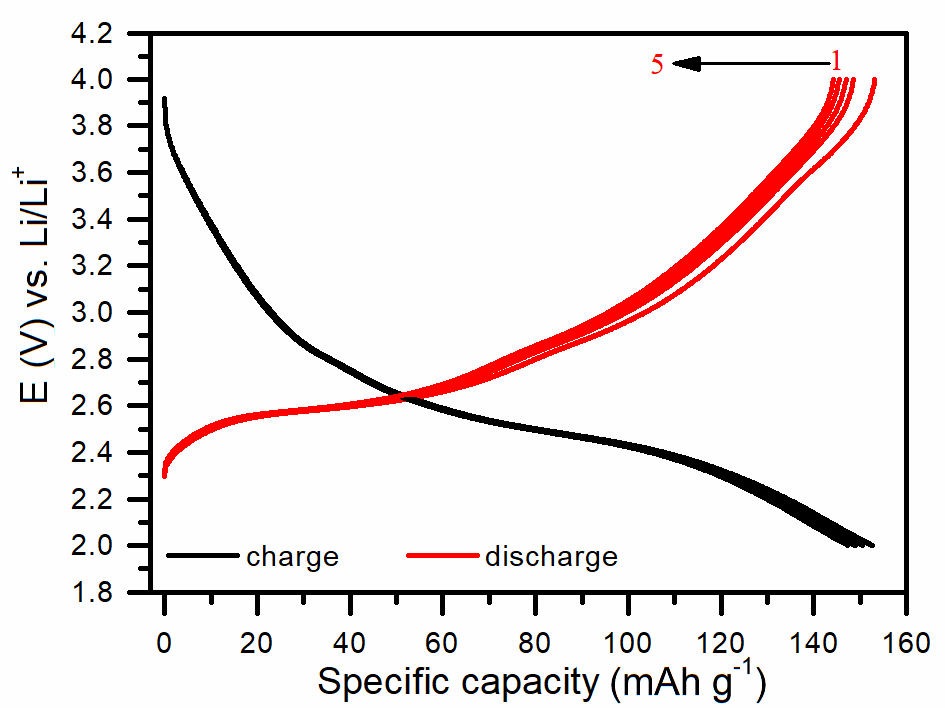**  b) |
| --- | --- |

**Figure S12**. Galvanostatic charge/discharge profiles of electrode materials after extended cycling at 1 A g^-1^in the 2.0 – 4.0 potential range (a) KVO-20 and (b) 1 KVO-40. The applied current density j = 100 mA g^-1^.


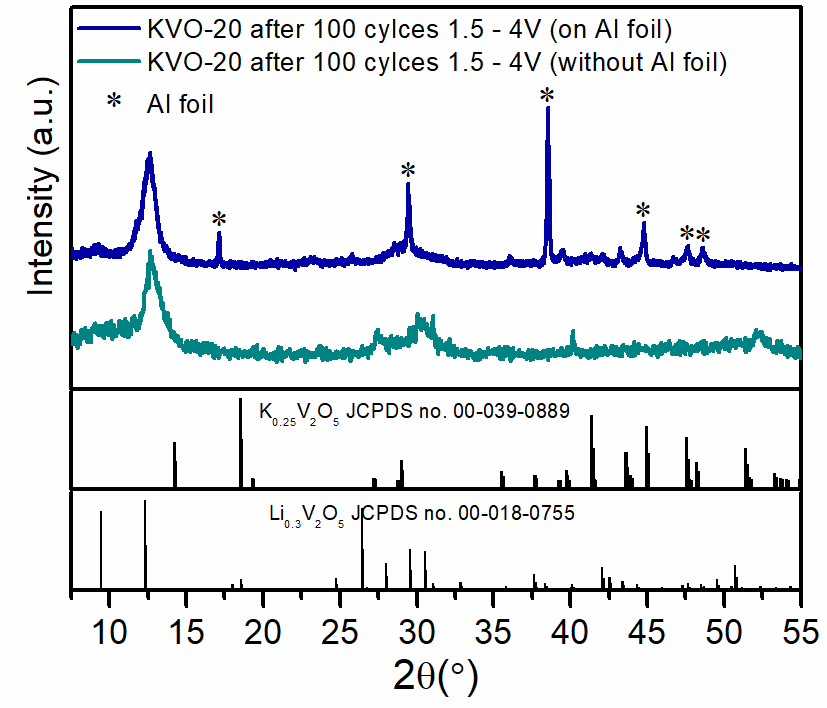


**Figure S13.** The representative XRD patterns after 100 cycles. KVO-20 electrode material on Al foil (blue line) and KVO-20 electrode material collected from Al foil (cyan line).

**Table S2**. The element ratio from the XPS analysis.

| Sample | KVO-20 | | | KVO-40 | | |
| --- | --- | --- | --- | --- | --- | --- |
|  | Pristine | 2 – 4 V | 1.5 – 4 V | Pristine | 2 – 4 V | 1.5 – 4 V |
| Cycles  Atomic ratio | - | 100 | 100 | - | 100 | 100 |
| V^4+^/V^5+^ | 0.10 | 0.20 | 0.40 | 0.02 | 0.40 | 0.40 |

**Table S3**. The element ratio from the MP-AES analysis.

| Voltage  range | Cycles | KVO-20 | | KVO-40 | |
| --- | --- | --- | --- | --- | --- |
|  |  | Atomic ratio | | | |
|  |  | V/K | V/Li | V/K | V/Li |
| 2 – 4 V | 100 | 5.33 | 4.70 | 4.22 | 5.50 |
| 1.5 – 4 V | 100 | 6.89 | 1.40 | 5.29 | 1.62 |

| Material | Voltage range (V) | Capacity (mAhg^-1^) | Current density (mA g^-1^) | Cycles | Ref. |
| --- | --- | --- | --- | --- | --- |
| K_0.25_V_2_O_5_ nanobelts | 1.5 – 4.0 | 120 | 1000 | 800 | ^59^ |
| K_0.5_V_2_O_5_ nanorods | 2.0 – 4.0 | 200 | 100 | 70 | ^63^ |
| K_0.25_V_2_O_5_ nanowires  KV_3_O_8_ nanowires | 1.5 – 4.0 | 172  292  178  301 | 1000  100  1000  100 | 500  100  500  100 | ^61^ |
| KV_3_O_8_ nanorods | 1.5 – 4.0 | 130 | 15 | 20 | ^60^ |
| K_0.25_V_2_O_5_ microspheres | 1.5 – 4.0 | 249  215  190 | 100  300  500 | 30  100  500 | ^60^ |
| K_0.66_V_3_O_8_ nanobelts | 1.5 – 4.0 | 197 | 75 | 25 | ^64^ |
| KVO-20  (K_2_V_6_O_16_·0.65H_2_O nanobelts) | 2.0 – 4.0 | 164  224 | 1000  100 | 100  5 | This work |
|  | 1.5 – 4.0 | 142  260 | 1000  100 | 100  5 | This work |
| KVO-40  (K_2_V_6_O_16_·0.76H_2_O nanobelts) | 2.0 – 4.0 | 164  170 | 1000  100 | 100  5 | This work |
|  | 1.5 – 4.0 | 179  260 | 1000  100 | 100  5 | This work |

**Table S4**. The electrochemical performance of cathode materials based on potassium vanadium oxides for LiBs
